# Association of daily physical activity and leisure-time exercise with dysphagia risk in community-dwelling older adults: a cross-sectional study

**DOI:** 10.1038/s41598-023-37605-z

**Published:** 2023-07-05

**Authors:** Tomoko Maehara, Rumi Nishimura, Akari Yoshitake, Mineko Tsukamoto, Yuka Kadomatsu, Yoko Kubo, Rieko Okada, Mako Nagayoshi, Takashi Tamura, Asahi Hishida, Kenji Takeuchi, Kenji Wakai, Mariko Naito

**Affiliations:** 1 Department of Oral Epidemiology, Graduate School of Biomedical and Health Sciences, Hiroshima University, 1-2-3 Kasumi, Minami-ku, Hiroshima, Hiroshima 734-8553 Japan; 2 Department of Public Oral Health, Graduate School of Biomedical and Health Sciences, Hiroshima University, 1-2-3 Kasumi, Minami-ku, Hiroshima, Hiroshima 734-8553 Japan; 3 Division of Dentistry and Oral Surgery, Japan, Community Health Care Organization, Tokuyama Central Hospital, 1-1 Kodacho, Shunan, Yamaguchi 745-0822 Japan; 4 Department of Preventive Medicine, Nagoya University Graduate School of Medicine, 65 Tsurumaicho, Showa-ku, Nagoya, Aichi 466-8550 Japan

**Keywords:** Geriatrics, Public health

## Abstract

This study aimed to clarify the association of daily physical activity and leisure-time exercise with the risk of dysphagia in community-dwelling Japanese older adults using a questionnaire-based survey. We analyzed 3070 participants (1657 men, 1413 women; age 66 ± 4 years [mean ± SD]) of the Shizuoka and Daiko studies within the Japanese Multi-Institutional Collaborative Cohort study. We used the Dysphagia Risk Assessment for the Community-dwelling Elderly questionnaire to assess dysphagia risk and the International Physical Activity Questionnaire to assess daily physical activity and leisure-time exercise. Logistic regression analyses were used to evaluate the independent association of the amount of physical activity and leisure-time exercise with dysphagia risk. The proportion of participants with dysphagia risk was 27.5% (n = 844) and the risk was significantly higher in women (29.8%, n = 421) than in men (25.5%, n = 423; *P* = 0.008). Daily physical activity was not associated with dysphagia risk. A greater amount of leisure-time exercise was associated with lower dysphagia risk (*P* for trend = 0.003) and individuals in the highest leisure-time exercise quartile had a significantly lower odds ratio (0.68, 95% CI 0.52–0.89) than those in the lowest quartile, even after adjusting for the covariates.

## Introduction

The frequency of dysphagia increases with age and 15–34% of community-dwelling older adults worldwide have dysphagia^1,2^. Dysphagia is associated with medical conditions such as stroke, neurological disease, dementia, head and neck cancer, and respiratory disorders. Risk factors for dysphagia among community-dwelling older adults include lifestyles such as reduced activities of daily living, malnutrition, depressive tendencies, age, medications, and chronic diseases^1–3^. Recovery after these diseases is difficult and preventing entering a frail state before the condition worsens is thus important^4^.

In Japan, long-term prevention programs are implemented to prevent frailty^4^. Community-based health programs for Japanese older adults combine oral health guidelines, physical exercise, and nutritional guidance^5^. Previous studies have shown that frailty is associated with the risk of declining oral function, including chewing and swallowing problems, which leads to malnutrition^6–8^. The early detection of dysphagia risk and prevention of frailty and subsequent diseases before a dysphagia diagnosis is therefore important for older adults to live healthy lives^7,9^.

Other studies have suggested that a decline in social activity may directly lead to a decline in oral function because of reduced human interaction and conversation^10,11^. Based on these studies, we believe that a low amount of physical activity and oral dysfunction may arise simultaneously. In addition, dysphagia risk is not limited to frail older adults. It is also important to detect the risk of dysphagia in the transition to frailty. However, few studies have found an association between the amount of physical activity and dysphagia.

Furthermore, there are various types and intensities of physical activity. It has been reported that leisure-time exercise reduces the risk of cardiovascular disease and all-cause mortality^12^. On the other hand, occupational physical activity does not confer health benefits because of low intensity or psychological stress (low work control) and other factors^13^. Different types and intensities of physical activity may be related to a different risk of disease, and spontaneous and non-spontaneous physical activities may differ. Therefore, the effects of daily physical activity and leisure-time exercise should be examined separately.

Additionally, although dysphagia is usually diagnosed using swallowing video endoscopy, video fluoroscopic examination, and various oral function tests, questionnaires have been reported as an easier and safer method to screen community-dwelling individuals at risk of dysphagia^14^. While questionnaire-based swallowing assessments do not diagnose dysphagia, they do assess conditions that predate dysphagia. Similarly, physical activity questionnaires can be used to assess such activity in a simplified way and can screen many people. Based on the above background, the present study aimed to clarify the association of daily physical activity and leisure-time exercise with the risk of dysphagia in community-dwelling Japanese older adults using a questionnaire-based survey.

## Methods

### Study participants

The data for the present study were obtained from the participants of the Shizuoka study and the Daiko study. These two studies are part of the long-term Japan Multi-Institutional Collaborative Cohort study conducted at 13 institutions to clarify the gene–environment interaction to prevent cancer and other lifestyle-related diseases^15^. The details of this study have been described in previous reports^16–19^.

In the Shizuoka study, the baseline survey was conducted from January 2006 to December 2007. The study participants (35–69 years) were recruited from health checkup examinees at the Seirei Preventive Health Care Center in Hamamatsu City, Shizuoka Prefecture, Japan. Of the 13,740 eligible individuals, 5040 (36.7%) participated in this study^16,18^.

**Figure 1:**
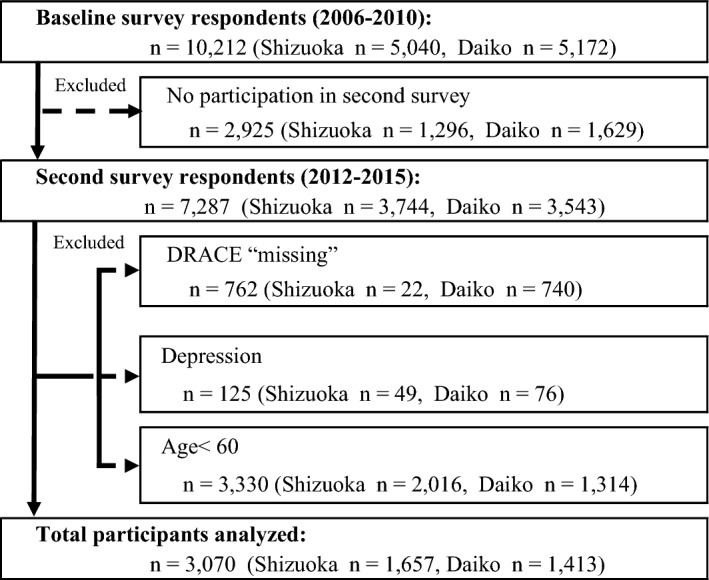
Flowchart of the participant recruitment process.

In the Daiko study, participants (35–69 years) were enrolled from the registered residents of Nagoya city. Residents were notified of the study mainly through the mailbox distribution of leaflets, personal communication, and local information such as via posters in public and commercial facilities. The survey was conducted at the Daiko Medical Center of Nagoya University in Nagoya, Japan from June 2008 to May 2010. After distributing 2,216,900 leaflets throughout Nagoya, 5172 participants were recruited^17,19^.We used the data of the second survey conducted in the Shizuoka study (2012–2013) and Daiko study (2014–2015)^16–19^ because the baseline survey did not include the DRACE questionnaire. From the baseline survey data, those who did not participate in the second survey and those who discontinued participation or withdrew their consent (2925; 1296 in Shizuoka, 1629 in Daiko) were excluded. Of the 7287 participants, those with missing values on the Dysphagia Risk Assessment for the Community-dwelling Elderly (DRACE) questionnaire (762; 22 in Shizuoka and 740 in Daiko) and those who suffer from depression (125; 49 in Shizuoka and 76 in Daiko) were excluded. In addition, those younger than 60 years (3330; 2016 in Shizuoka and 1314 in Daiko) were excluded because DRACE is a reliable and valid questionnaire for older people. Subsequently, 3070 (1657 men and 1413 women; age: 66.5 ± 4.2 years [mean ± SD], 60–76 years) who responded to the questionnaire were included in the analysis. The flowchart of the participant recruitment process is presented in Fig. 1.

### Questionnaire

The participants were asked to complete a self-administered questionnaire that included questions on dysphagia risk and lifestyle. The questionnaire developed for the Shizuoka study and Daiko study was used^16–21^. Information on medical histories; medication; lifestyle characteristics, including smoking status, alcohol consumption, sleep duration, and dietary behavior over the past year; and daily physical activity and leisure-time exercise was obtained. Those who suffer from depression were assessed through self-reported medication of antipsychotics, including antidepressants and anxiolytics. These participants were excluded because their subjective judgment may be unclear and depression was strongly associated with dysphagia in this study. We did not conduct cognitive function tests; however, the participants lived independently and participated in health checkups by themselves. Furthermore, all the participants could answer the questionnaire and provide written informed consent. All the responses were collected by trained staff.

### Assessment of daily physical activity and leisure-time exercise

We used the questionnaire to quantify daily physical activity and leisure-time exercise. The activities were estimated based on the International Physical Activity Questionnaire (long version)^22^, a versatile and widely used questionnaire in epidemiological studies worldwide, including Japan. The questionnaire in this study was similar to those used in the Japan Public Health Center-based prospective study; the validity of the amount of physical activity estimated from the questionnaire results was demonstrated^23^. Daily physical activities were grouped into four categories according to activity level. These were activities that involve (i) using muscle power, (ii) walking, (iii) standing, and (iv) sitting during daily work, housework, and commuting to work, with metabolic equivalent (MET) values of 4.5, 3.0, 2.0, and 1.5, respectively. The duration of each activity was classified into eight categories (assigned average hours per day in parentheses): almost none (0), < 1 (0.5), 1 to < 3 (2), 3 to < 5 (4), 5 to < 7 (6), 7 to < 9 (8), 9 to < 11 (10), and ≥ 11 (11) h/day. The amount of daily physical activity was expressed in MET–h/week for each activity ([MET level] × [hours of activity per day] × 7), and the total amount was calculated by summing these values over the three higher levels. Sitting was excluded because of its low energy expenditure (≈1.5 METs) and association with various health problems^24^. It was also excluded from a previous study evaluating daily physical activity^25^. Sitting time was thus calculated separately from daily physical activities and expressed as MET–h/week ([MET level 1.5] × [hours of activity per day] × 7).

Leisure-time exercise was divided into three MET levels: light intensity (e.g., walking and hiking), moderate intensity (e.g., light jogging and swimming), and vigorous intensity (e.g., marathon running and combat sports)^22^. Similar to the assessment of daily physical activity, total leisure-time exercise was expressed in MET–h/week for each of these three levels. In these estimations, METs of 3.4, 7.0, and 10.0 were assigned to light, moderate, vigorous intensity, respectively. The duration of each activity was classified into six categories (assigned average hours per event in parentheses): < 30 min (15/60), 30 to < 60 min (45/60), 1 to < 2 h (1.5), 2 to < 3 h (2.5), 3 to < 4 h (3.5), and ≥ 4 h (4). Frequency per week was classified into five categories (assigned average times per day in parentheses): almost none (0), 1–3 times/month (2/30), 1–2 times/week (1.5/7), 3–4 times/week (3.5/7), and 5 times/week or over (6/7).

### Assessment of dysphagia risk

Dysphagia risk was evaluated using the DRACE questionnaire^6^. DRACE includes 12 items, with a score from 0 to 2 assigned to each item: the total DRACE score thus ranges from 0 to 24^26^. A total DRACE score of ≥ 4 indicates a risk of dysphagia for community-dwelling older people^27^, and this cutoff value was used in our study. DRACE is a useful tool with sufficient reliability and validity in the Japanese population for detecting the latent risk of chewing and swallowing disorders among frail community-dwelling older adults^6^.

### Statistical analyses

Continuous variables were calculated as mean ± standard deviation. However, those with skewed distributions were expressed as medians (25th and 75th percentiles). Categorical variables were calculated using numbers and percentages. The *t*-test, Wilcoxon rank-sum test, and chi-square test were used to compare the characteristics between the risk of dysphagia categories (yes or no), sexes, and amount of leisure-time exercise groups where appropriate.

The association of daily physical activity and leisure-time exercise with the risk of dysphagia was evaluated using logistic regression analyses. The adjusted covariates were as follows: age (years, continuous) and sex (men and women combined only) for Model 1; age, sex (men and women combined only); research sites (Shizuoka or Daiko); education level (≤ 9, 10–15, ≥ 16 years, or unknown); smoking habit (current, past, or never smokers or unknown); alcohol drinking status (current drinkers or other or unknown); energy intake (kcal/day, continuous); Body Mass Index (BMI; < 18.5, 18.5 to < 25, ≥ 25, or unknown); working time (h/week), sleep duration (< 6, 6 to < 7, 7 to < 8, or ≥ 8 h/day), medical history of cancer, cerebrovascular disease, or heart disease for the multivariate-adjusted model.

Daily physical activity (MET–h/week, quartiles) and leisure-time exercise (MET–h/week, quartiles) were simultaneously added into all the models. The linear association of daily physical activity or leisure-time exercise with the risk of dysphagia was statistically tested by assigning ordinal variables of 1, 2, 3, and 4 to the quartiles of activity in the logistic regression analyses and the results were converted into trend *P* values. These results were also shown separately by sex because there are differences in sex such as muscle strength, smoking habit, alcohol consumption, and diseases associated with dysphagia such as oral cancer and chronic obstructive pulmonary disease^28,29^.

For daily physical activity, we also analyzed the sitting time (calculated in MET–h/week quartiles) and the risk of dysphagia, adjusting for the multivariate variables used in the logistic regression analysis. Moreover, we performed stratified analyses on the associations between leisure-time exercise and the risk of dysphagia in groups with or without a medical history of cancer, cerebrovascular disease, or heart disease in the logistic regression analysis and measured the trend *P* values. This is because those being treated for cancer, cerebrovascular disease, or heart disease may have been unable to perform physical activities or may have had difficulty eating or swallowing. In addition, for each item in DRACE, logistic regression analysis was performed. All the logistic regression analyses were performed adjusting for multivariate variables. All the calculations and statistical tests were performed using SPSS software (version 27 for Windows; IBM, Armonk, NY). All the statistical tests were two-sided and *P* < 0.05 was considered to be statistically significant.

### Ethics approval and consent to participate

The study received ethical approval from the ethics committee of the Nagoya University Graduate School of Medicine, Japan (approval numbers 2008-0618-4 and 2008-0618-5) and the Ethical Committee for Human Genome Research of Hiroshima University, Japan (approval numbers Gen-253 and Gen-254). The study was performed in conformity with the Declaration of Helsinki and regulations in Japan. Informed consent was obtained from all the participants before any procedures commenced.

## Results

The mean (SD) age of the participants was 66.5 (4.2) years (60–76) for men and 66.5 (4.1) years (60–76) for women; there was no significant difference in age (*P* = 0.522). The average DRACE score increased with an increase in age. The average score by age group was 2.2 ± 2.3 (men 2.2 ± 2.3, women 2.3 ± 2.4,) for 60–64 years, 2.7 ± 2.6 (men 2.5 ± 2.6, women 2.8 ± 2.5,) for 65–74 years, and 3.3 ± 2.8 (men 3.7 ± 3.1, women 2.7 ± 2.1) for 75–76 years. The proportion of the participants with dysphagia risk was 27.5% (n = 844), significantly higher among women (29.8%, n = 421) than among men (25.5%, n = 423; *P* = 0.008).

The mean (SD) amount of daily physical activity was 121.3 (93.6) (MET–h/week) for men and 156.3 (82.5) (MET–h/week) for women. The mean (SD) amount of leisure-time exercise was 22.9 (27.3) (MET–h/week) for men and 20.5 (24.7) (MET–h/week) for women. For leisure-time exercise, the mean (SD) for light intensity was 106.7 (123.3) (MET–h/week) for men and 86.3 (106.6) (MET–h/week) for women. The mean (SD) for moderate intensity was 48.3 (123.6) (MET–h/week) for men and 56.2 (119.6) (MET–h/week) for women. The mean (SD) for vigorous-intensity was 9.2 (60.1) (MET–h/week) for men and 4.9 (44.0) (MET–h/week) for women.

The participant characteristics according to the risk of dysphagia by sex are shown in Table 1. Those participants with dysphagia risk were older, had a shorter working time (h/week), and worked less than those without dysphagia risk for both sexes. Men with dysphagia risk had a shorter sleep duration than those without dysphagia risk. Moreover, women with dysphagia risk had a lower energy intake than those without dysphagia risk. Additionally, we compared our participants’ characteristics with those of the general Japanese population using official statistical data from the Health Service Bureau, National Health and Nutrition Survey, National Cancer Registry in Japan, and Patient Survey by the Ministry of Health, Labour and Welfare^30–32^. We found that 34.8% of our participants had a final education level of junior college/university graduate or above, which was higher than that of the general population (16.8%). Of our participants, 92.9% were non-smokers, which was higher than among the general population (79.6%); 44.8% were non-drinkers, which was lower than among the general population (59.4%); and 16.7% were obese, which was lower than among the general population (29.1%). Among the participants, 7.0% had diabetes, 9.8% had cancer, 3.0% had cerebrovascular disease, and 5.0% had heart disease, which were all lower than among the general population (22.9%, 16.0%, 13.1%, and 20.4%, respectively).

**Table 1:** Characteristics of the study participants according to the risk of dysphagia by sex.

	Men		*P*	Women		*P*
	Risk of dysphagia			Risk of dysphagia		
	No	Yes		No	Yes	
	(n = 1234)	(n = 423)		(n = 992)	(n = 421)	
Age (years)^a^	66.3 (4.1)	67.0 (4.1)	0.001	66.3 (4.2)	67.3 (4.2)	< 0.001
Energy intake (kcal/day)^b^	1849.0 (1677.4, 2011.7)	1827.9 (1656.5, 1999.4)	0.784	1478.6 (1329.1, 1596.1)	1494.8 (1355.9, 1634.5)	0.048
BMI (n, %)			0.691			0.887
< 18.5	30 (2.4)	13 (3.1)		115 (11.6)	50 (11.9)	
18.5 to < 25	954 (77.3)	318 (75.2)		741 (74.7)	327 (77.7)	
≥ 25	245 (19.9)	90 (21.3)		134 (13.5)	44 (10.5)	
Unknown	5 (0.4)	2 (0.5)		2 (0.2)	0 (0)	
Sleep duration (h/day)^a^	7.0 (1.0)	6.8 (1.0)	0.010	6.5 (0.9)	6.4 (1.0)	0.090
Daily physical activity^b^ (MET–h/week)	98.0 (49.0, 168.0)	101.5 (54.3, 169.8)	0.538	155.8 (85.8, 203.0)	155.8 (106.8, 203.0)	0.556
Leisure-time exercise (MET–h/week)^b^	14.3 (4.8, 29.8)	14.3 (3.6, 28.6)	0.129	14.3 (3.6, 28.6)	12.6 (3.57, 28.6)	0.403
Working time (h/week)^b^	19.6 (21.2)	16.2 (20.4)	0.004	9.9 (16.1)	7.1 (13.6)	0.002
Working	683 (55.3)	201 (47.5)	0.007	372 (37.5)	126 (29.9)	0.008
Education level (years) (n, %)			0.727			0.728
≤ 9	117 (9.5)	47 (11.1)		106 (10.7)	38 (9.0)	
10–15	656 (53.2)	209 (162)		762 (76.8)	341 (81.0)	
≥ 16	451 (36.5)	162 (38.3)		117 (11.8)	41 (9.7)	
Unknown	10 (0.8)	5 (1.2)		7 (0.7)	1 (0.2)	
Smoking habit (n, %)			0.701			0.172
Current	132 (10.7)	40 (9.5)		23 (2.3)	18 (4.3)	
Past	704 (57.1)	256 (60.5)		60 (6.0)	29 (6.9)	
Never	397 (32.2)	126 (29.8)		908 (91.5)	371 (88.1)	
Unknown	1 (0.1)	1 (0.2)		1 (0.1)	3 (0.7)	
Current alcohol drinking (n, %)			0.622			0.476
Yes	905 (73.3)	305 (72.1)		333 (33.6)	150 (35.6)	
No	329 (26.7)	118 (27.9)		658 (66.3)	270 (64.1)	
Unknown	0 (0.0)	0 (0.0)		1 (0.1)	1 (0.2)	
Prevalence (n, %)^c^						
Cancer	129 (10.5)	50 (11.8)	0.435	77 (7.8)	45 (10.7)	0.073
Cerebrovascular disease	37 (3.0)	20 (4.7)	0.092	23 (2.3)	13 (3.1)	0.401
Heart disease	75 (6.1)	31 (7.3)	0.364	29 (2.9)	17 (4.0)	0.280

^a^Mean (SD); ^b^Median (25%, 75%); ^c^Cancer, cerebrovascular disease, or heart disease: self-reported by questionnaire.

An analysis of the characteristics of each leisure-time exercise group by sex revealed that both men and women had significant differences in age (men and women *P* < 0.001), sleep duration (men *P* = 0.002, women *P* = 0.010), and working time (men and women *P* < 0.001). Among men, significant differences were observed for smoking habit (*P* = 0.001), and among women, significant differences were observed for a medical history of cancer (*P* = 0.048).

As shown in Table 2, no significant difference was observed between the amount of daily physical activity and the risk of dysphagia after adjusting for all the covariates. We also analyzed the sitting time and DRACE values adjusting for the covariates used in the logistic regression analysis but found no significant association (*P* = 0.680).

**Table 2:** Risk of dysphagia according to the amount of daily physical activity (MET–h/week).

Daily physical activity (MET–h/week)	Q1 ≤ 64.8	Q2 64.9–113.8	Q3 113.9–183.8	Q4 ≥ 183.9	*P* for trend
Men and women (n = 3070)					
No. of participants	720 (23.5)	757 (24.7)	791 (25.8)	802 (26.1)	
No. of dysphagia risk cases (n, %)	1343 (23.2)	124 (27.8)	36 (30.3)	16 (31.4)	
Age- and sex-adjusted OR^a^ (95% CI)	1.00 (Ref.)	1.11 (0.87–1.41)	1.26 (0.99–1.60)	1.07 (0.84–1.36)	0.489
Multivariate-adjusted OR^b^	1.00 (Ref.)	0.98 (0.76–1.25)	0.97 (0.75–1.25)	0.82 (0.63–1.06)	0.122
Men (n = 1657)					
No. of participants	546 (33.0)	441 (26.6)	327 (19.7)	343 (20.7)	
No. of dysphagia risk cases (n, %)	134 (24.5)	111 (25.2)	93 (28.4)	85 (24.8)	
Age-adjusted OR^c^ (95% CI)	1.00 (Ref.)	1.05 (0.78–1.41)	1.23 (0.90–1.69)	1.01 (0.74–1.39)	0.646
Multivariate-adjusted OR^b^ (95% CI)	1.00 (Ref.)	0.92 (0.68–1.25)	0.99 (0.70–1.38)	0.84 (0.60–1.18)	0.409
Women (n = 1413)					
No. of participants	174 (12.3)	316 (22.4)	464 (32.8)	459 (32.5)	
No. of dysphagia risk cases (n, %)	44 (25.3)	95 (30.1)	150 (32.3)	132 (28.8)	
Age-adjusted OR^c^ (95% CI)	1.00 (Ref.)	1.28 (0.84–1.96)	1.39 (0.93–2.08)	1.20 (0.80–1.80)	0.605
Multivariate-adjusted OR^b^ (95% CI)	1.00 (Ref.)	1.09 (0.70–1.69)	1.01 (0.65–1.56)	0.83 (0.53–1.31)	0.209

*CI* confidence interval, *OR* odds ratio.

^a^Age- and sex-adjusted OR: age, sex, the amount of leisure-time exercise (quartile).

^b^Multivariate-adjusted OR: age, sex (men and women combined only), energy intake, BMI, sleep duration, working time, education level, smoking habit, current alcohol drinking, medical history of cancer/cerebrovascular disease/heart disease, and study area, the amount of leisure-time exercise (quartiles).

^c^Age-adjusted OR: age, the amount of leisure-time exercise (quartile).

As shown in Table 3, a greater amount of leisure-time exercise was associated with a lower risk of dysphagia for all participants (*P* for trend = 0.003). The individuals in the highest leisure-time exercise quartile had a considerably lower odds ratio (0.68, 95% CI 0.52–0.89) than those in the lowest quartile, even after adjusting for all the covariates.

**Table 3:** Risk of dysphagia according to the amount of leisure-time exercise (MET–h/week).

Leisure-time exercise (MET–h/week)	Q1 ≤ 1.79	Q2 1.80–8.92	Q3 8.93–21.63	Q4 ≥ 21.64	*P* for trend
Men and women (n = 3070)					
No. of participants	417 (13.6)	830 (27.0)	752 (24.5)	1071 (34.9)	
No. of dysphagia risk cases (n, %)	130 (31.2)	233 (28.1)	203 (27.0)	278 (26.0)	
Age- and sex-adjusted OR^a^ (95% CI)	1.00 (Ref.)	0.86 (0.67–1.12)	0.78 (0.60–1.02)	0.70 (0.55–0.91)	0.004
Multivariate-adjusted OR^b^	1.00 (Ref.)	0.84 (0.65–1.10)	0.77 (0.58–1.01)	0.68 (0.52–0.89)	0.003
Men (n = 1657)					
No. of participants	196 (11.8)	451 (27.2)	417 (25.2)	593 (35.8)	
No. of dysphagia risk cases (n, %)	58 (29.6)	119 (26.4)	105 (25.2)	141 (23.8)	
Age-adjusted OR^c^ (95% CI)	1.00 (Ref.)	0.84 (0.58–1.22)	0.75 (0.51–1.10)	0.67 (0.46–0.96)	0.021
Multivariate-adjusted OR^b^ (95% CI)	1.00 (Ref.)	0.83 (0.57–1.22)	0.73 (0.49–1.08)	0.65 (0.44–0.95)	0.019
Women (n = 1413)					
No. of participants	221 (15.6)	379 (26.8)	335 (23.7)	478 (33.8)	
No. of dysphagia risk cases (n, %)	72 (32.6)	114 (30.1)	98 (29.3)	137 (28.7)	
Age-adjusted OR^c^ (95% CI)	1.00 (Ref.)	0.87 (0.61–1.25)	0.80 (0.55–1.16)	0.74 (0.52–1.06)	0.088
Multivariate-adjusted OR^b^ (95% CI)	1.00 (Ref.)	0.84 (0.57–1.22)	0.79 (0.53–1.17)	0.71 (0.48–1.03)	0.074

*CI* confidence interval, *OR* odds ratio.

^a^Age- and sex-adjusted OR: age, sex, the amount of daily physical activity (quartiles).

^b^Multivariate-adjusted OR: age, sex (men and women combined only), energy intake, BMI, sleep duration, working time, education level, smoking habit, current alcohol drinking, medical history of cancer/cerebrovascular disease/heart disease, and study area, the amount of daily physical activity (quartiles).

^c^Age-adjusted OR: age, the amount of daily physical activity (quartiles).

Men also demonstrated a low odds ratio (0.65, 95% CI 0.44–0.95) in the highest leisure-time exercise quartile, and a greater amount of leisure-time exercise was associated with a lower risk of dysphagia (*P* for trend = 0.019), while women demonstrated borderline significance after adjusting for all the covariates (*P* for trend = 0.074).

The amount of leisure-time exercise performed by those with a medical history of cancer, cerebrovascular disease, or heart disease was analyzed and no significant difference was observed between the amount of leisure-time exercise and the risk of dysphagia after adjusting for all the covariates. Men showed significantly lower odds ratios in the other quartiles than in the lowest quartile. On the other hand, women showed a borderline significant trend toward a higher risk of dysphagia as physical activity levels increased (*P* for trend = 0.055). The analysis of the amount of leisure-time exercise performed by those without a history of cancer, cerebrovascular disease, or heart disease demonstrated that the highest leisure-time exercise quartile had a low odds ratio (0.66, 95% CI 0.49–0.89). Women also had a low odds ratio (0.59, 95% CI 0.40–0.89) in the highest leisure-time exercise quartile, while men in this quartile had borderline significance (*P* for trend = 0.068) and a low odds ratio (0.76, 95% CI 0.49–1.86) after adjusting for all the covariates except medical histories (Table 4).

**Table 4:** Risk of dysphagia according to the amount of leisure-time exercise with and without medical history.

Leisure-time exercise (MET–h/week)		Q1 ≤ 1.79	Q2 1.80–8.92	Q3 8.93–21.63	Q4 ≥ 21.64	*P* for trend
With cancer, cerebrovascular disease, or heart disease	Men and women (n = 511)					
	No. of participants	63 (12.3)	138 (27.0)	124 (24.3)	186 (36.4)	
	No. of dysphagia risk cases (n, %)	23 (36.5)	36 (26.1)	39 (31.5)	63 (33.9)	
	Age- and sex-adjusted OR^a^ (95% CI)	1.00 (Ref.)	0.59 (0.31–1.12)	0.77 (0.40–1.47)	0.83 (0.45–1.54)	0.681
	Multivariate-adjusted OR^b^	1.00 (Ref.)	0.49 (0.25–0.97)	0.64 (0.32–1.28)	0.72 (0.37–1.39)	0.975
	Men (n = 313)					
	No. of participants	36 (11.5)	72 (23.0)	78 (24.9)	127 (40.6)	
	No. of dysphagia risk cases (n, %)	17 (47.2)	16 (22.2)	21 (26.9)	36 (28.3)	
	Age-adjusted OR^c^ (95% CI)	1.00 (Ref.)	0.30 (0.13–0.72)	0.37 (0.16–0.85)	0.38 (0.17–0.84)	0.138
	Multivariate–adjusted OR^b^ (95% CI)	1.00 (Ref.)	0.21 (0.08–0.55)	0.26 (0.10–0.66)	0.29 (0.12–0.70)	0.105
	Women (n = 198)					
	No. of participants	27 (13.6)	66 (33.3)	46 (23.3)	59 (29.8)	
	No. of dysphagia risk cases (n, %)	6 (22.2)	20 (30.3)	18 (39.1)	27 (45.8)	
	Age-adjusted OR^c^ (95% CI)	1.00 (Ref.)	1.45 (0.50–4.15)	2.06 (0.69–6.19)	2.53 (0.86–7.43)	0.052
	Multivariate-adjusted OR^b^ (95% CI)	1.00 (Ref.)	1.62 (0.48–5.44)	2.14 (0.62–7.44)	2.90 (0.85–9.89)	0.055
Without cancer, cerebrovascular disease, or heart disease	Men and women (n = 2559)					
	No. of participants	354 (13.8)	692 (27.0)	628 (24.5)	885 (34.6)	
	No. of dysphagia risk cases (n, %)	107 (30.2)	197 (28.5)	164 (26.1)	215 (24.3)	
	Age- and sex-adjusted OR^a^ (95% CI)	1.00 (Ref.)	0.92 (0.70–1.23)	0.78 (0.58–1.04)	0.68 (0.51–0.90)	0.001
	Multivariate-adjusted OR^d^	1.00 (Ref.)	0.91 (0.68–1.22)	0.78 (0.58–1.05)	0.66 (0.49–0.89)	0.001
	Men (n = 1344)					
	No. of participants	160 (11.9)	379 (28.2)	339 (25.2)	466 (34.7)	
	No. of dysphagia risk cases (n, %)	41 (25.6)	103 (27.2)	84 (24.8)	105 (22.5)	
	Age-adjusted OR^c^ (95% CI)	1.00 (Ref.)	1.08 (0.70–1.64)	0.91 (0.59–1.41)	0.77 (0.50–1.17)	0.059
	Multivariate-adjusted OR^d^ (95% CI)	1.00 (Ref.)	1.07 (0.69–1.65)	0.90 (0.57–1.40)	0.76 (0.49–1.86)	0.068
	Women (n = 1215)					
	No. of participants	194 (16.0)	313 (25.8)	289 (23.8)	419 (34.5)	
	No. of dysphagia risk cases (n, %)	66 (34.0)	94 (30.0)	80 (27.7)	110 (26.3)	
	Age-adjusted OR^c^ (95% CI)	1.00 (Ref.)	0.82 (0.56–1.21)	0.70 (0.47–1.04)	0.62 (0.43–0.91)	0.010
	Multivariate-adjusted OR^d^ (95% CI)	1.00 (Ref.)	0.80 (0.54–1.19)	0.70 (0.46–1.07)	0.59 (0.40–0.89)	0.009

*CI* confidence interval, *OR* odds ratio.

^a^Age- and sex-adjusted OR: age, sex, the amount of daily physical activity (quartiles).

^b^Multivariate-adjusted OR: age, sex (men and women combined only), energy intake, BMI, sleep duration, working time, education level, smoking habit, current alcohol drinking, medical history of cancer/cerebrovascular disease/heart disease, and study area, the amount of daily physical activity (quartiles).

^c^Age-adjusted OR: age, the amount of daily physical activity (quartiles).

^d^Multivariate-adjusted OR: age, sex (men and women combined only), energy intake, BMI, sleep duration, working time, education level, smoking habit, current alcohol drinking, and study area, the amount of daily physical activity (quartiles).

The items in the DRACE questionnaire associated with a decreased risk of dysphagia, as the amount of leisure-time exercise increased were the “sensation of food or liquid rising into the throat from the stomach” (*P* for trend < 0.001), “taking a long time to eat” (*P* for trend = 0.003), “difficulties with chewing hard food” (*P* for trend = 0.001), “difficulties with swallowing” (*P* for trend = 0.023), and “choking during a meal” (*P* for trend = 0.012) (Tables 5, S1).

**Table 5:** Risk of DRACE each item according to the amount of leisure-time exercise (MET–h/week).

DRACE items	Q1 ≤ 1.79	Q2 1.80–8.92	Q3 8.93–21.63	Q4 ≥ 21.64	*P* for trend
No. of participants (%)	417 (13.6)	830 (27.0)	752 (24.5)	1071 (34.9)	
Multivariate-adjusted OR^a^ (95% CI)					
Get fever	1.00 (Ref.)	1.08 (0.75–1.56)	0.98 (0.67–1.43)	0.87 (0.60–1.27)	0.236
Taking a long time to eat	1.00 (Ref.)	0.95 (0.72–1.25)	0.95 (0.71–1.26)	0.68 (0.51–0.91)	0.003
Difficulties with swallowing	1.00 (Ref.)	0.82 (0.60–1.12)	0.63 (0.45–0.87)	0.71 (0.52–0.97)	0.023
Difficulties with chewing hard food	1.00 (Ref.)	0.81 (0.63–1.03)	0.84 (0.65–1.08)	0.66 (0.52–0.84)	0.001
Food falling from the mouth	1.00 (Ref.)	0.74 (0.54–1.00)	0.74 (0.54–1.01)	0.83 (0.62–1.13)	0.616
Choking during a meal	1.00 (Ref.)	1.12 (0.87–1.44)	0.98 (0.75–1.27)	0.81 (0.63–1.05)	0.012
Choking when swallowing liquid	1.00 (Ref.)	0.91 (0.70–1.18)	0.95 (0.72–1.24)	0.86 (0.66–1.12)	0.326
Food rising into the nasal cavity	1.00 (Ref.)	0.62 (0.35–1.12)	0.81 (0.45–1.44)	0.64 (0.36–1.14)	0.334
Hoarseness after meals	1.00 (Ref.)	0.82 (0.42–1.60)	0.97 (0.50–1.89)	0.78 (0.40–1.51)	0.583
Expectoration of sputum during meals	1.00 (Ref.)	1.14 (0.75–1.73)	0.94 (0.61–1.47)	1.13 (0.74–1.71)	0.797
Sensation of food being stuck in the esophagus	1.00 (Ref.)	0.94 (0.69–1.29)	0.99 (0.72–1.37)	0.75 (0.54–1.03)	0.058
Sensation of food or liquid rising into the throat from the stomach	1.00 (Ref.)	0.79 (0.61–1.01)	0.62 (0.48–0.81)	0.64 (0.49–0.82)	< 0.001

*CI* confidence interval, *OR* odds ratio.

^a^Multivariate-adjusted OR: age, sex, energy intake, BMI, sleep duration, working time, education level, smoking habit, current alcohol drinking, medical history of cancer/cerebrovascular disease/heart disease, and study area, the amount of daily physical activity (quartiles).

## Discussion

This study examined the association of the risk of dysphagia with daily physical activity and leisure-time exercise in 3,070 community-dwelling Japanese older adults using a questionnaire-based survey. Daily physical activity and leisure-time exercise were evaluated separately. Among the community-dwelling Japanese older adults, an inverse association was found between the amount of leisure-time exercise and the risk of dysphagia, and a greater amount of leisure-time physical exercise was associated with a lower risk of dysphagia.

The proportion of the participants with dysphagia risk in this study was 27.5%; previous studies have demonstrated that 15–35% of community-dwelling older adults have dysphagia^1,2^. Moreover, Miura et al. reported that the mean DRACE score for subjects aged 65–74 years is 2.4 ± 2.6^33^, which is comparable with the value in the present study (3.3 ± 2.8).

The sex-stratified analysis demonstrated a more significant trend among women, while no association was found between the amount of daily physical activity and the risk of dysphagia. In this study, women had a higher risk of dysphagia than men. It is assumed that this is because men had higher levels of education than women. People with higher levels of education tend to have higher oral functioning^8^ and more leisure-time in which to exercise^13^. Although the DRACE questionnaire was found to be valid concerning swallowing function^6^, swallowing problems are subjective and women are more aware of problems related to chewing and swallowing in their daily lives than men. For example, women indicated a higher percentage of chewing and swallowing problems in the National Health and Nutrition Survey^34^.

Our results showed that men demonstrated a low odds ratio in the highest leisure-time exercise quartile, while women demonstrated a borderline significant association in this quartile. The results among those without medical histories showed similar associations. In the group with cancer, cerebrovascular disease, or heart disease, the results in women were opposite to the overall result. Background factors for changes in health intention and behavior are related to medical histories^35^ and this may increase the amount of exercise performed.

In this study, the risk of dysphagia was low in the highest leisure-time exercise quartile. In experiments on rats, running has been found to be associated with muscle strength related to the tongue and breathing^36^. Another study demonstrated an association between swallowing muscles and trunk muscle mass in healthy older adults^37^. Additionally, it has been found that trunk training strengthens respiratory muscle movement, affects the swallowing reflex, and accentuates breathing. It may therefore be useful to recommend exercise to prevent the development of dysphagia.

No association was found between the amount of daily physical activity and the risk of dysphagia. However, the risk of dysphagia tended to decrease in the groups with higher amounts of leisure-time exercise compared with the groups with lower amounts of leisure-time exercise. Leisure-time exercise differs from daily physical activity in that the former involves dedicating time to exercise. When considering the load of METs, which indicates the amount of leisure-time exercise, the METs coefficient was 7.0–10.0 (moderate to vigorous). For daily physical activity, the METs coefficient was 3.0–4.5 (walking, using muscle power)^22^. The METs coefficient indicates how many times the load is applied compared with 1 at rest. Leisure-time exercise is considered to be more than twice as heavy as daily physical activity. Exercises with a METs above 6 are defined as activities that cause sweating and a change in heart and respiratory rates. Furthermore, resistance exercises are especially important to improve muscle mass and strength^38^. As many such exercises are performed during leisure-time exercise, this may account for the finding of no significant difference in the risk of dysphagia in daily physical activity.

From another point of view, Nagayoshi et al.^39^ found that leisure activities are associated with higher tongue pressure, suggesting that interacting with people may be one way of maintaining oral function. Older Japanese individuals tend to prefer exercising with others; increasing the frequency of exercising with others may thus have a positive health impact^40^. Conversing, laughing, and eating require oral function and may improve or maintain tongue muscles and the respiratory system. The participants in the group with the highest amount of leisure-time exercise had a decreased risk of dysphagia. They exercised more than 21.64 METs per week; thus, one criterion could be that exercising more than 22 METs per week might decrease the risk of dysphagia. According to a report by the Ministry of Health, Labour and Welfare of Japan, it is recommended that people between 18 and 64 years perform physical activity with an intensity of 3 METs or more, at 23 METs/week^41^; our result is equivalent to that. Internationally, all adults should undertake 150–300 min of moderate-intensity or 75–150 min of vigorous-intensity physical activity per week, or an equivalent combination thereof^41^.

Among the DRACE items, the sensation of food or liquid rising into the throat from the stomach, taking a long time to eat, difficulties with chewing hard food, and difficulties with swallowing, and choking during a meal were associated with the risk of dysphagia. Only the sensation of food or liquid rising into the throat from the stomach differed significantly. People with gastroesophageal reflux may avoid vigorous exercise and strength training because of esophageal acid exposure because strength training increases acid reflux^42^. Gastric acid reflux and exercise are associated with and difficult to distinguish from dysphagia; however, in this study, we could not assess or exclude from our data those diagnosed with gastroesophageal reflux disease.

We identified an association between physical activity and the risk of dysphagia in a large sample of community-dwelling Japanese adults. In addition, our participants were healthier and less affected by medical conditions than inpatients and institutional residents. Although the demographic data indicated that the study population was healthier than general Japanese adults between 60 and 70 years, the prevalence of dysphagia was within the generally indicated range^1,2^.

However, several limitations should be considered. First, we could not prove a causal relationship because of the cross-sectional design of this study. Second, the participants in the Shizuoka study were health checkup examinees and all participated voluntarily in the cohort study. They may thus be more health-conscious than the general population. Their better health status may have had positive effects on their daily physical activities and exercise habits. Third, we could not adjust for all diseases and oral functions related to dysphagia, such as chewing ability, swallowing function, gastroesophageal reflux disease, and chronic obstructive pulmonary disease^26,38^. These individuals may have a high risk of dysphagia, physical disabilities, or poor nutrition; hence, they may partake in less physical activities than those in the present study. They should prioritize nutritional management^43^ or other treatments than exercise. Finally, physical activity and dysphasia were assessed with a self-administered questionnaire. The present results are underestimated, and if evaluated using objective indicators, those at risk for dysphagia may be more prevalent^44^.

## Conclusions

The present study suggests that while daily physical activities are not associated with dysphagia risk, those who perform more leisure-time exercise are at a lower risk of dysphagia than those who perform less leisure-time exercise. The results of this study suggest that in contrast to dysphagia caused by disease or disability, dysphagia caused by physical inactivity may require the performance of leisure-time exercise to maintain social interaction and muscle strength.

## Supplementary Information

Supplementary Table S1.

## Data Availability

The datasets used and analyzed in the current study are available from the corresponding author upon reasonable request and approval of the ethics committee.

## Abbreviations

MET: Metabolic equivalent
DRACE: Dysphagia Risk Assessment for the Community-dwelling Elderly
BMI: Body Mass Index
