# Supplementary material for: Association of daily physical activity and leisure-time exercise with dysphagia risk in community-dwelling older adults: a cross-sectional study
Source: Sci Rep. 2023 Jul 5;13:10893. doi: 10.1038/s41598-023-37605-z (PMC10322846; doi:10.1038/s41598-023-37605-z)
Supplement: Supplementary file 1 — Supplementary Table S1. [file 41598_2023_37605_MOESM1_ESM.pdf]

Supplemental Table S1. Percentage of the risk with DRACE each item according to the amount of leisure-time exercise (MET–h/ week)

**DRACE items**	**Q1 ≤ 1.79**			**Q2 1.80–8.92**			**Q3 8.93–21.63**			**Q4 ≥ 21.64**		
No. of participants (%)	417 (13.6)			830 (27.0)			752 (24.5)			1071 (34.9)		
	Men	Women	Total	Men	Women	Total	Men	Women	Total	Men	Women	Total
Get fever	28 (11.1)	36 (13.5)	64 (12.3)	54 (15.8)	33 (12.2)	87 (14.2)	42 (9.0)	50 (12.4)	92 (10.6)	53 (8.9)	55 (11.6)	108 (10.1)
Taking a long time to eat	61 (24.2)	72 (27.0)	133 (25.6)	73 (21.4)	77 (28.5)	150 (24.5)	111 (23.7)	106 (26.4)	217 (24.9)	114 (19.2)	114 (24.1)	228 (21.4)
Difficulties with swallowing	40 (15.9)	62 (23.2)	102 (19.7)	43 (12.6)	55 (20.4)	98 (16.0)	68 (14.5)	56 (13.9)	124 (14.3)	81 (13.6)	87 (18.4)	168 (15.7)
Difficulties with chewing hard food	106 (42.1)	120 (44.9)	226 (43.5)	139 (40.8)	118 (43.7)	257 (42.1)	177 (37.8)	182 (45.3)	359 (41.3)	213 (35.9)	190 (40.2)	403 (37.8)
Food falling from the mouth	62 (24.6)	43 (16.1)	105 (20.2)	59 (17.3)	47 (17.4)	106 (17.3)	77 (16.5)	57 (14.2)	134 (15.4)	120 (20.2)	76 (16.1)	196 (18.4)
Choking during a meal	84 (33.3)	94 (35.2)	178 (34.3)	112 (32.8)	113 (41.9)	225 (36.8)	144 (30.8)	146 (36.3)	290 (33.3)	170 (28.6)	158 (33.4)	328 (30.7)
Choking when swallowing liquid	67 (26.6)	89 (33.3)	156 (30.1)	99 (29.0)	82 (30.4)	181 (29.6)	129 (27.6)	127 (31.6)	256 (29.4)	160 (26.9)	149 (31.5)	309 (29.0)
Food rising into the nasal cavity	15 (6.0)	11 (4.1)	26 (5.0)	12 (3.5)	8 (3.0)	20 (3.3)	23 (4.9)	14 (3.5)	37 (4.3)	27 (4.5)	16 (3.4)	43 (4.0)
Hoarseness after meals	10 (4.0)	11 (4.1)	21 (4.0)	11 (3.2)	8 (3.0)	19 (3.1)	20 (4.3)	8 (2.0)	28 (3.2)	23 (3.9)	10 (2.1)	33 (3.1)
Expectoration of sputum during meals	30 (11.9)	22 (8.2)	52 (10.0)	36 (10.6)	20 (7.4)	56 (9.2)	38 (8.1)	32 (8.0)	70 (8.0)	67 (11.3)	34 (7.2)	101 (9.5)
Sensation of food being stuck in the esophagus	36 (14.3)	65 (24.3)	101 (19.5)	58 (17.0)	63 (23.3)	121 (19.8)	62 (13.2)	79 (19.7)	141 (16.2)	64 (10.8)	90 (19.0)	154 (14.4)
Sensation of food or liquid rising into the throat from the stomach	96 (38.1)	95 (35.6)	191 (36.8)	115 (33.7)	80 (29.6)	195 (31.9)	122 (26.1)	119 (29.5)	241 (27.7)	155 (26.1)	141 (29.8)	296 (27.7)
